# Supplementary material for: High microbial diversity in glacial habitats uncoupled from the specialized microbiomes of resident chironomid fauna
Source: PLoS One. 2026 Jan 8;21(1):e0340651. doi: 10.1371/journal.pone.0340651 (PMC12782408; doi:10.1371/journal.pone.0340651)
Supplement: S1 File — (DOCX) [file pone.0340651.s001.docx]

**Supporting Information**


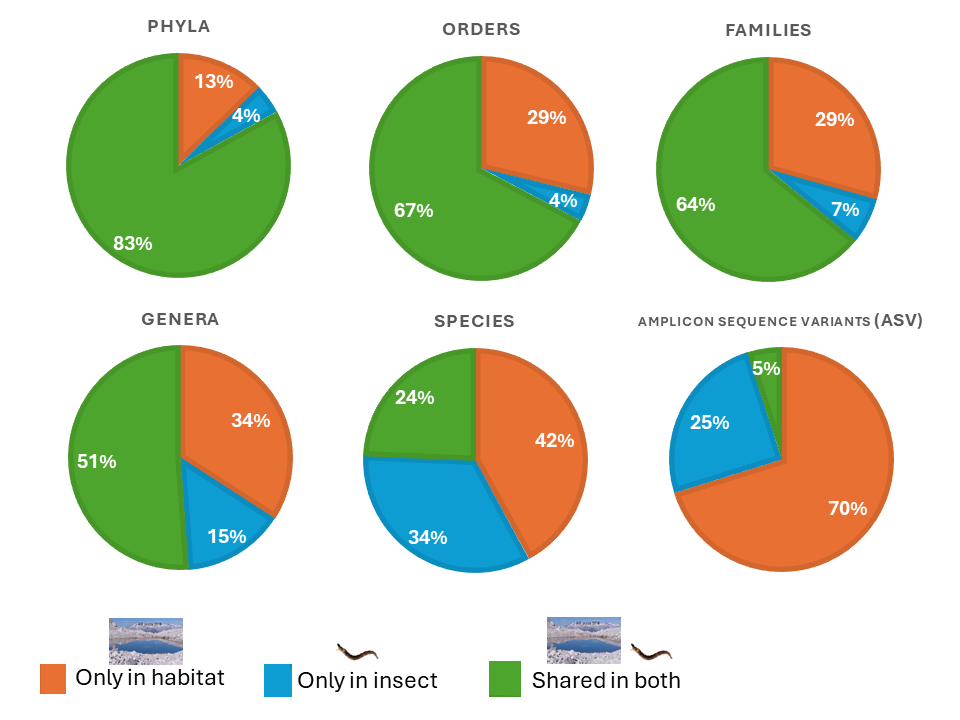


**Fig. S1.** **Pie chart representation of the extent of commonality vs. specificity percent reversal upon constraining the taxonomical resolution towards its deepest level.** Percent values are rounded up to integers

Coherence of the insect-habitat partitioning ratios between single glacier vs. three glaciers datasets

Due to the differently represented proportion of habitats vs. insects in the different glaciers we plotted the proportions of the resulting microbiota, at the broadest (phylum) and finest (ASV) taxonomical resolution level, comparing the results of the single Agola glacier with those from the three glaciers together The trends were very similar as shown in the following image, supporting the validity of the inferences drawn from the analyses.

**Fig. S2. Comparison of the percentages of Phyla and ASVs (Amplicon Sequence Variants), occurring in the habitat only, or shared between it and the insects, or found just in the insects.** The trends of the sole Agola glacier are plotted along with those of all three glaciers' data together,


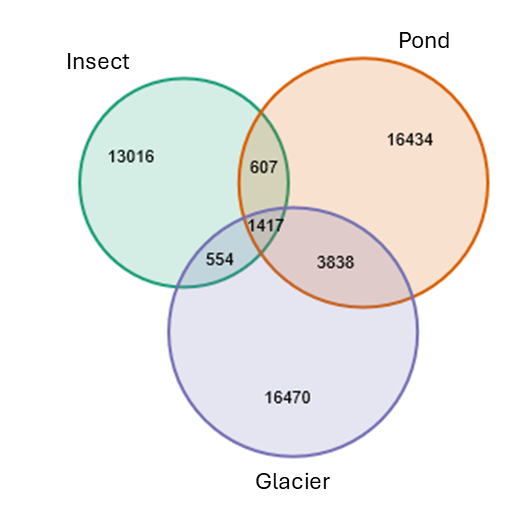


**Fig. S3**. **Venn Diagram of the shared and unique numbers of ASV among insects, pond and glacier**

| **Ecological concept** | **Suggested mechanism of microbial support** | **Specific ecological insight** |
| --- | --- | --- |
| Resilience to glacial melt | Metabolic Stability and Detoxification: Glacial melt introduces environmental instability, including fluctuating flow rates, temperature spikes, and chemical shifts. The highly specific core microbiome acts as a physiological buffer. Taxa like *Providencia* and *Serratia* (core insect taxa) are functionally known in insect systems for their roles in detoxification and breaking down complex or inhibitory compounds, ensuring metabolic continuity even when the ingested detrital quality rapidly changes due to melt events. | This stable internal community can confer resistance to environmental stress, allowing the chironomids to maintain essential physiological functions where a generalist community would fail to cope with fluctuations typical of glacier-fed streams. |
| Colonization of new environments | Nutrient Supplementation (The 'Symbiotic Propagule'): The lack of significant difference in microbial community structure across ten chironomid species and three distant glacier sites underscores the perspective importance of the core microbiome. This core community could entail also a vital symbiotic propagule that the host has an advantage in carrying with it. | By supplying essential nutrients in a consistent manner, such as nitrogen or complex carbon resources, this core could facilitate the rapid and successful colonization of newly deglaciated, oligotrophic, primary succession habitats. The host-symbiont pairing seems pre-adapted to the nutrient-scarce conditions inherent in these environments. |

**Tab. S1.** **ecological implications expected for the microbiota of the investigated envirooinment**

| **Dominant core taxon** | **Inferred ecological role in chironomid gut** | **Insights** |
| --- | --- | --- |
| *Providencia, Serratia* (Gammaproteobacteria) | Nutrient Scavenging and Detritivory: These genera are commonly associated with insect guts and are known for their high metabolic versatility, including the breakdown of complex organic compounds. In the low-quality, detritus-rich diet of the chironomid, they are likely key primary decomposers, providing the host with readily absorbable organic acids and amino acids. | Their prevalence suggests a dedicated function in maximizing energy and nutrient extraction from a nutritionally poor food source, which is crucial for survival in the oligotrophic glacial stream environment. |
| *Massilia* (Betaproteobacteria) | Cold-Adaptation and Immune Support: *Massilia* members are frequently psychrotolerant (cold-enduring) and have been implicated in various insect-microbe interactions. They likely contribute to host fitness by maintaining robust metabolic activity at the low temperatures typical of glacier-fed streams. | Their role is inferred to be one of physiological support, maintaining gut homeostasis and potentially participating in defense or signaling under extremely cold conditions. |
| *Flavobacterium* (Bacteroidetes) | Polymer Degradation (e.g., Chitin): Members of the Bacteroidetes phylum are renowned for their massive arsenal of glycoside hydrolases. *Flavobacterium* is likely selected for its capacity to break down large, tough biopolymers, such as the chitin found in fungal debris or the cell walls of microbial mats that form part of the chironomid's scraped or ingested diet. | This is a highly specific enzymatic service required to release sequestered nutrients and energy that the chironomid host could not obtain otherwise, further suggesting a direct role in nutrition. |

**Tab. S2.** **Ecologically insightful roles of insect-specific core taxa**

| **Dominant environmental taxon/group** | **Inferred ecological role in habitat** | **Insights** |
| --- | --- | --- |
| *Arenimonas* | Primary Colonization and Nutrient Mineralization: Often found in proglacial sediments and newly deglaciated soils. | This genus is a key pioneer colonizer, capable of metabolizing diverse organic compounds and mineralizing phosphorus and nitrogen, which facilitates the early stages of ecosystem development. |
| *Methylotenera* | Carbon Cycling (Methylotrophy): These are specialized bacteria that utilize one-carbon compounds (e.g., methanol, methylamine). | They act as critical carbon processors in the habitat, often forming syntrophic relationships that mitigate the release of methane or process specialized carbon sources available in glacial meltwater. |
| Nitrosomonadaceae | Nitrogen Cycling: , members of this family are recognized chemoautotrophs, specifically involved in nitrite oxidation (a key step in nitrification). | Their role solidifies the glacier habitat as a site of intense, externally-driven nitrogen transformation, but the lack of strong overlap with the insect gut suggests that the chironomid's core microbiome is selected to bypass reliance on this external cycle. |
| Vicinamibacterales | Sorption and Adherence: This order often includes taxa associated with soil and sediment, likely serving a role in binding and forming the microbial mats and biofilms from which the chironomid larvae forage. | Their high prevalence highlights the structural microbial component of the glacial environment, which forms the physical substrate that the chironomids scrape or ingest, but their limited presence in the gut suggests effective host selection/filtering. |

**Tab. S3.** **Ecological Insights into Environment-Dominant Taxa**
