# Supplementary material for: High microbial diversity in glacial habitats uncoupled from the specialized microbiomes of resident chironomid fauna
Source: PLoS One. 2026 Jan 8;21(1):e0340651. doi: 10.1371/journal.pone.0340651 (PMC12782408; doi:10.1371/journal.pone.0340651)
Supplement: Attachment — Submitted filename: Answers to Editor and Reviewers.docx [file pone.0340651.s003.docx]

PONE-D-25-16248

Journal Requirements:

When submitting your revision, we need you to address these additional requirements.

1. Please ensure that your manuscript meets PLOS ONE's style requirements, including those for file naming. The PLOS ONE style templates can be found at

<https://journals.plos.org/plosone/s/file?id=wjVg/PLOSOne_formatting_sample_main_body.pdf> and [https://journals.plos.org/plosone/s/file?id=ba62/PLOSOne_formatting_sample_title_authors_affiliations.pdf](https://track.editorialmanager.com/CL0/https:%2F%2Fjournals.plos.org%2Fplosone%2Fs%2Ffile%3Fid=ba62%2FPLOSOne_formatting_sample_title_authors_affiliations.pdf/1/010f0197e3a04bb3-f4feb70a-1e63-40fd-a861-d14e3a2b13f4-000000/FarZuc4trbU0e7scCzoZEYEz4ycwr-agq3xBtLPg7jA=218)

ANSWER: The text format has been uniformed to the required templates.

2. In your Methods section, please provide additional information regarding the permits you obtained for the work. Please ensure you have included the full name of the authority that approved the field site access and, if no permits were required, a brief statement explaining why.

ANSWER: The following information has been added: “The permit to sample from the site has been released by Dr. Cristiano Trotter, the Director of Parco Naturale Adamello Brenta, Via Nazionale, 24, 38080 Strembo (TN).”

3. Thank you for stating the following financial disclosure:

[Grant BioMiti 2018, issued from Adamello-Brenta Natural Park, Italy, awarded to A.Z.].

Please state what role the funders took in the study.  If the funders had no role, please state: "The funders had no role in study design, data collection and analysis, decision to publish, or preparation of the manuscript."

If this statement is not correct you must amend it as needed.

Please include this amended Role of Funder statement in your cover letter; we will change the online submission form on your behalf.

ANSWER: The following statement has been added: "The funders had no role in study design, data collection and analysis, decision to publish, or preparation of the manuscript."

The amended role of the funder has been stated in the cover letter.

3. Thank you for stating the following in the Acknowledgments Section of your manuscript:

[This work was made possible in part by funding from the Adamello-Brenta Natural Park, Project Grant BioMiti 2018. Alessandra Franceschini is gratefully acknowledged for technical support and logistic assistance during the expedition.]

We note that you have provided funding information that is currently declared in your Funding Statement. However, funding information should not appear in the Acknowledgments section or other areas of your manuscript. We will only publish funding information present in the Funding Statement section of the online submission form.

Please remove any funding-related text from the manuscript and let us know how you would like to update your Funding Statement. Currently, your Funding Statement reads as follows:

[Grant BioMiti 2018, issued from Adamello-Brenta Natural Park, Italy, awarded to A.Z.].

Please include your amended statements within your cover letter; we will change the online submission form on your behalf.

ANSWER: We have removed the funding-related text from the Acknowledgments as requested, and indicated it in the cover letter.

4. We note that Figure 1 in your submission contains map/satellite images which may be copyrighted. All PLOS content is published under the Creative Commons Attribution License (CC BY 4.0), which means that the manuscript, images, and Supporting Information files will be freely available online, and any third party is permitted to access, download, copy, distribute, and use these materials in any way, even commercially, with proper attribution. For these reasons, we cannot publish previously copyrighted maps or satellite images created using proprietary data, such as Google software (Google Maps, Street View, and Earth). For more information, see our copyright guidelines: <http://journals.plos.org/plosone/s/licenses-and-copyright>.

We require you to either (1) present written permission from the copyright holder to publish these figures specifically under the CC BY 4.0 license, or (2) remove the figures from your submission:

ANSWER: The mentioned image in Fig. 1 has been substituted with a non-satellite physical map.

**Reviewer #1**:

The manuscript presents an interesting investigation into the microbial ecology of glacial systems, focusing on the comparison between environmental bacterial communities and those associated with chironomids inhabiting glacial environments. The research addresses an important and little-explored topic, particularly with regard to the possible relationships between host-associated microbiomes and those present in the surrounding extreme environment. The topic is certainly of interest to a wide scientific audience, particularly in the fields of microbial ecology and environmental biology.

ANSWER: We acknowledge these appreciative comments

However, the manuscript requires substantial revisions to improve clarity, text organisation and consistency between the various sections, as well as to justify certain methodological choices and interpretations more effectively. A revision of the English is essential. More scientific and direct English is needed, with simple and more schematic sentences. Along the entire manuscript, all sentences can be made simpler and clearer. Once the structure and content have been finalised, I recommend a review by a native English speaker.

ANSWER: The structure of the manuscript, along with its language and style aspects, has been thoroughly revised. The version showing the highlighted changes in yellow will be useful to appreciate the capillary editing that has been applied throughout the paper.

However, a major gap in this work is the lack of statistical data to support the findings and the lack of images that clearly represent the objectives of the work (e.g., NMDS for community overlap between hosts and environment, Venn diagrams to determine how many ASVs, species or genera are shared, etc.). These aspects need to be integrated.

ANSWER: As will be specifically highlighted below, the statistical analyses (NMDS ordination plot, PERMANOVA, ANOSIM, PERMDISP) have been performed, and the results, showing the significant p values for the separation of each sample category, have been added in the new fig. 4. A Venn diagram (Fig. S3, Supplementary information) and Jaccard distances (text) have been added as well.

Below are detailed comments by section and specific suggestions line by line to help the authors strengthen their work.

Introduction
The introduction is grammatically correct, with a few minor exceptions listed below. However, I find the writing style overly complex. The language is not very scientific; it seems more ‘poetic’ and, in a context where simple and direct concepts need to be expressed for a good understanding of the scientific context, it is distracting and confusing. There are also very long sentences that slow down readability, or sentences that seem to have been translated word for word. Here are some examples:

1) Line 54: ‘In spite of the concept of low temperatures as keepers of life stillness...’. It does not clearly explain the concept of low temperatures blocking biological activity. Why not write something like: ‘Despite the common perception of low temperatures as preserving biological inactivity...’? This is just one example. Another is line 72: ‘As a destiny of deglaciated foreland...’. It would be better to write more directly.

ANSWER: The suggestions are sound, and sentences have been accordingly revised.

2) Line 60: ‘Meta-analyses have evidenced sets of functions...’. ‘Evidenced’ seems to have been translated directly. It would be better to use ‘identified’, ‘highlighted’, ‘showed’, etc. Line 70: ‘An inspection of the prokaryotic benthic diversity...’. Use ‘analysis’ or ‘assessment’ instead of ‘inspection’.

ANSWER: Correction made (highlighted). The second sentence is no longer present as suggested in a subsequent query.

3) Lines 79-83 express a simple concept but are written in an overly complex manner and require multiple readings. They could be simplified with ‘Glacial environments also host a variety of invertebrate animals, which interact closely with microbial communities. In these animals, microbes are primarily found in the gut and may be either resident, contributing to physiological or symbiotic functions, or transient, originating from ingested food.’

ANSWER: The text has been revised as advised.

There are also unnecessary additions, such as ‘as in any other context’ (line 80), and non-scientific definitions, such as ‘The glacial setting’ (line 79). Glaciers are not settings but environments.

ANSWER: The unnecessary words as well as the ‘glacial setting’ expression, are no longer in the text as the sentence has been replaced by the one suggested in the previous query.

Another strange construction: ‘communicative interplay between the two dimensions of the outer environment and the inner microbiome’ (lines 80-81).

ANSWER: That sentence has also been removed because of the above text substitution.

Another example lines 108-110: “Chironomids from cold habitats have also been studied in relation to their feeding using carbon-stable isotopes. In this respect, the past glacier dynamics were found to be the main driver of autochthonous primary productivity”. This could be replaced with ‘Carbon stable isotope studies have shown that feeding patterns in chironomids are influenced by past glacier dynamics, which shape autochthonous primary productivity.’

ANSWER: The text has been revised as suggested.

The text also contains sentences such as ‘Covering almost 10% of the earth's surface, glaciers offer a valuable ground for comparative research, from which meta-analyses have evidenced sets of functions with significant recurrence, among which nitrogen fixation, aerobic nitrite oxidation and nitrification’ (lines 59-61) and ‘An inspection of the prokaryotic benthic diversity comparing glacial and non-glacial streams indicated a higher richness for the latter’ (lines 70-71), which are not expanded upon in the following sections and do not seem to serve much purpose in the introduction. In the first case, the functions mentioned are not reconsidered for further investigation. In the second case, non-glacial areas have never been mentioned before, so why mention this comparison?

ANSWER: We agree, and both sentences have been removed.

Finally, I think that changing the order of the topics in the introduction could enhance the work. The focus is on chironomids and how their microbiota compares with that of the external environment. So, I would start with chironomids and their presence in glacial environments, and why they are better subjects than other organisms for this type of study -> intestinal microbiota -> glacial environments and environment-insect links in these extreme environments. This is just a suggestion, but generally, the introduction needs to improve its flow and establish much stronger and more direct connections between the cited topics.

ANSWER: The order of topics has been restructured as suggested.

56 melted water -> melted ice

ANSWER: Correction made.

60 from which, meta-analyses -> remove the comma

ANSWER: That sentence has been removed in the restructured introduction.

62 phyla as -> phyla such as

ANSWER: Correction made.

63 genera as -> genera such as

ANSWER: Correction made.

65 Ferruginibacter genus have been also signaled -> Ferruginibacter genus have been also reported? Observed?

ANSWER: Correction made.

88-89 even the often neglected dimension of individual variation, is the source… -> even the often-neglected individual variation is the (remove dimension of and comma)

ANSWER: Correction made.

99 offsprings -> offspring

ANSWER: Correction made.

113 at characterizing -> to characterize

ANSWER: Correction made.

Materials and Methods

127-132 Is there any additional information on the sites for a more detailed description? Average temperatures and precipitation, exposure, any data that can be used to describe the sites and assess whether they are similar or different, and to contextualise the results obtained.

ANSWER: A table (new Table 1, shown below) has been added reporting all the available information. Regarding meteorological data, there are no permanent meteorological stations around the three sites.

**Tab. 1. Physico-chemical properties of the sampling sites.** The data refer to the sampling dates presented in Tab. 2, with the exception of the water temperature (Mean Tw_max) whose value refers to the mean of the 3 weeks before the sampling, collected by dataloggers. Q: water discharge (absent in Agola being it a static pond and not a flowing kryal stream).

| Sampling site | Agola (pond) | Mandrone (kryal) | Amola (kryal) |
| --- | --- | --- | --- |
| Altitude site (m asl) | 2596 | 2569 | 2540 |
| Distance from the source (km) | 0.02 | 0.05 | 0.14 |
| Suspended sediments (mg/l) | 2.60 | 76.80 | 131.00 |
| Mean Tw_max (°C) | 4.5 ± 1.5 | 1.2 ± 0.5 | 1.6 ± 0.4 |
| Chlorophyll a (µg/cm2) | 0.09 | 0.48 | 0.33 |
| pH (20°C) | 8.20 | 6.9 | 6.6 |
| Conductivity (μS/cm) | 72.0 | 5.5 | 13.4 |
| SiO2 (mg/l) | 0.3 | 1.1 | 1.2 |
| Q (m^3^/s) | - | 3.8 | 1.1 |

132 Remove comma between Agola coordinates and Fig citation.

ANSWER: Correction made.

148-151 wherever necessary, what does it mean? It is better to split the sentence and explain better, like: “Chironomid larvae were collected using a 100-µm mesh pond net. Taxonomic identification was initially done via a morphological approach (microscopic examination of larval features). When morphology was not enough for an appropriate identification, we performed molecular identification by extracting the larval DNA and amplifying the mitochondrial cytochrome oxidase I (COI) gene, following the protocol of [25]”. Add somewhere the number of collected samples: e.g. 12 environmental samples, 34 larvae and 2 adults.

ANSWER: The text has been revised as suggested.

157 Remove the comma at the end of the subtitle.

ANSWER: Correction made.

151 and 159 The methods are too vague. Describe at least the kit used for the extraction and the primers.

ANSWER: The following text has been added: “Briefly, genomic DNA was extracted from the larval body using a QIAGEN DNAeasy Blood & Tissue Kit, following a slightly modified protocol for the purification of total DNA from animal tissue. In particular, 10 μl of a 1 M DTT (dithiothreitol) solution was added to enhance the removal of chitin, after which the samples were incubated overnight at 56 °C for the initial lysis step. The final elution was performed in 50 μl of AE buffer to increase the final DNA concentration. The mitochondrial (COI gene were amplified and sequenced using the primers LCO1490 GGTCAACAAATCATAAAGATATTGG and HCO2198 TAACTTCAGGGTGACCAAAAAATCA”.

159-164 One question: Did you include negative controls during DNA extraction (both for insects and the environment) to check for contamination? Low-biomass samples, such as glacier ice, can be prone to contamination from reagents, so it is common to perform blank extractions. If you did, please indicate how they were handled (and presumably no amplification in them). If you did not include them, this limitation could be worth acknowledging later. I recommend at least noting whether a blank was performed during the sequencing workflow and what the result was (hopefully, negligible reads). You may consider also this relevant paper that emphasizes the importance of including and sequencing negative controls in studies involving low-biomass alpine environments: <https://doi.org/10.1016/j.ecolind.2022.109737>, <https://doi.org/10.1016/j.scitotenv.2023.168159>.

ANSWER: Yes, in this type of studies we routinely perform negative controls using the same reagents (extraction without sample and eluate sequencing), and we also did those as custom, for the batches used in the series that includes the present experiments. Such tests consistently yield numbers of reads that are below the detection level as they do not pass the quality filtering bioinformatics cutoff.

We thank you for signalling the mentioned reference, which we have added and cited about the background minimization.

170 Remove comma after sets.

ANSWER: Correction made.

168-170 Explain the methodology better. Was the PCR program the same for both primer pairs? The two primer pairs were used together in a multiplex, or have you run two PCRs per sample? Were the pooled amplicons from the amplification of the two different primer pairs?

ANSWER: The multi-amplicon system does not adopt two primer pairs but two primer sets, which cover seven hypervariable regions (primer set V2-4-8 and primer set V3-6,7-9). The two PCR reactions are run separately, pooled afterwards, and use the same thermal program. Details have been added to the text as follows:

“The Kit is designed for multi-amplicon analyses of polybacterial samples using Ion Torrent™ sequencing technology. It includes two primer sets that selectively amplify seven hypervariable regions of the 16S region in bacteria in two separate reactions. The combination of the two primer pools allows for sequence-based identification of a broad range of bacteria within a mixed population.”

Have you run PCR replicates? They are important for low biomass samples like glacier water or small insect guts.

ANSWER: For the level at which replicates are deemed critical (biological or technical) we opted for the former, following the advice stemming from Rob Knighr’s seminal paper (Marotz et al., 2019 *Triplicate PCR reactions for 16S rRNA gene amplicon sequencing are unnecessary*, BioTechniques 67: 29-32 (July 2019) 10.2144/ btn-2018-0192. In which authors compared single PCR reactions to pooled triplicate PCR reactions for 16S rRNA gene amplicon sequencing

on nearly 400 samples from a diverse range of environments across three independent laboratories, and showed that, in spite of conventional concerns, modern amplicon data analysis employs

methods that beame less sensitive to previously critical artifacts. Their comparison of results from single versus triplicate reactions for 16S amplicon sequencing evidenced no significant impact of adopting a less labor-intensive single reaction protocol. Relying on this reassuring demonstration published by one of the front-leading groups in microbiome studies, we prioritized to allocate the sequencing run’s throughput to the representation of full biological replicates for the variables under investigation.

175 Xpress Plu 9… and Express Bacorde Kit. Which is correct? Xpress or Express?

ANSWER: The correct name is Xpress. Correction applied.

183 Expected reads length?

ANSWER: The average read length achieved by quality-passing outputs of this Ion sequencing chemistry is around 250, within a range spanning between 200 and 400 bases.

192 “the feature and taxonomy tables were exported” is not necessary.

ANSWER: The words have been removed.

200 Which model do you use on the SHAMAN online utility?

ANSWER: As this program was used only for the results presented in Fig. 3 we chose a three-variable model with the taxonomical depth set to the genus level, Data have been added to the text.

201 Sequences have been deposited in NCBI’s Sequence Read Archive under accession PRJNA1222183.

ANSWER: Correction made.

I would suggest adding statistical analyses to strengthen the interpretation of differences between microbial communities associated with insects and the environment. It would be appropriate to include a Non-metric Multidimensional Scaling (NMDS) analysis based on Bray–Curtis distances. This analysis would have provided an effective visual representation of community structure, highlighting any separation between groups. In addition, applying a statistical test such as PERMANOVA (adonis in R) would have allowed a quantitative assessment of whether the differences observed between chironomid species and sites were statistically significant. These approaches are standard in microbial ecology analyses and would have added statistical rigour and robustness to the conclusions. Furthermore, including a Venn diagram and similarity indices (e.g., Jaccard) between groups would have further supported the observations on the low community overlap. In any case, statistical analyses are necessary and fundamental for a correct interpretation of the differences.

ANSWER: We acknowledge this as a very relevant point and a major necessity for the manuscript. We have performed the statistical analysis recommended and added a new figure (Fig. 4) with the 3D ordination plot of the NMDS showing the separation between the insect- and environment-associated bacteria. The results were backed up by three statistical analyses (PERMANOVA, PERMDISP and ANOSIM) whose significant p-values are shown in the same novel figure.

In addition, we have constructed the Venn diagram as suggested and included it in the Supplementary Information file (Fig. S3).

We have likewise computed the suggested index and added the values in the text as follows.

“The Jaccard similarity index on the whole ASV data matrix means yielded the following values: insect vs. pond: 0.0564; insect vs. glacier: 0.0548; glacier vs. pond: 0.1336. As regards the Bray-Curtis distances those were: insect vs. pond: 0.9367; insect vs. glacier: 0.9412; glacier vs. pond: 0.5504.”

Results

The results are interesting, but I regret to say that this section needs to be completely revised. This part of the manuscript mixes results, discussion and even materials and methods (see lines 378-383, where it emerges that another analysis was performed). The results section must follow closely the materials and methods section. If the first paragraph of the materials and methods section discusses extraction and amplification, the results section must discuss what was obtained in terms of the quality of the results (e.g., all samples were extracted and amplified, or some samples yielded too low and were discarded), numerical data, and comparisons supported by statistical data. And so on, following the structure of the materials and methods. In addition, many descriptive parts should go in the discussion and not in the results. Furthermore, the sentences are complex and not very scientific. Here, you need to present the raw data, almost like a list.

ANSWER: We have dealt with the section considering the comments. As regards the mention of the sentence which described a method, it had not been anticipated in the M&M section, as it is solely a mathematical step, but we have now provided a description of it also in that part (“The differential representation of ASV’s between groups of samples was computed as log_2_ fold-change of the means.”).

Regarding the suggested order of the results presentation, we have verified the corresponding series of the topics in Materials and Methods and ensured that data on DNA yield and sequencing output would be in the first paragraph, and that the subsequent aspects would follow. We also noticed a certain asymmetry between the 9 paragraphs within the Results section and the subsequent single paragraph in the Discussion. Upon trying different rearrangements, we felt that the clearest solution was to combine the two sections in a single one (Results and Discussion), which is among the options that the journal allows. Therefore, we have restructured both and moved parts where appropriate to clear some redundancies and achieve a much more organized version of the manuscript.

284 Since you have Table 2, Figure 4 is not necessary. The table is more visually appealing.

ANSWER: The figure has been removed from the manuscript and placed in the Supplementary Information file. In our opinion, compared to the data shown in former Tab. 2 (now Tab. 3) it could have an added value in conveying in a single visual glance the evidence of the progressive reduction of insect plus habitat shared taxa along the deepening of taxonomy ranks. In any event, we are ready to drop it from there as well if Reviewers and Editor feel that appropriate.

Discussion

The discussion should be better organized following the main hypotheses. The discussion is weak and does not properly explore the results.

ANSWER: As anticipated, the combining of Results and Discussion addressed this aspect as well.

Eg. 460-474 Interesting but out of the objective of the manuscript.

ANSWER: Those lines have been removed.

476 This is not in contrast with… Try to be more direct in your sentences. The way they are structured now, they are convoluted and require multiple readings to be understood.

ANSWER: The litotes has been converted into an assertion (This is in line with…)

Eg. 486-490 The examples (quails, forests, air) are not directly relevant to insects or glacial environments. The paragraph attempts to justify that the host species and site did not influence the microbiome, but it does so by referring to very different systems with different biological contexts. It does not clearly link back to the main objective of the study: whether the gut microbes of chironomids reflect environmental communities or form distinct assemblages associated with the host.

ANSWER: We agree, also because there was a redundancy with a subsequent point of the discussion for some of those topics, which were repeated later. The mentioned lines have been cancelled. For the comment on the airborne microbiota, we have had a contrasting request since the other reviewer had instead asked to add a detail. However, that sentence has been moved to another part of the manuscript to avoid distracting from the main hypothesis discussion.

The main objectives of the paper are taken into consideration in the discussion, but they are treated marginally and get lost in digressions that are not strictly related to the objective of the work. Furthermore, works that have nothing to do with the context are often cited.

ANSWER: We removed the ancillary reasoning and drove the focus on the main objective. The last paragraph of Results and Discussion (*Overall considerations on the tested hypothesesis*) focused on it.

Be sure to explicitly circle back to the “symmetry vs specificity” hypothesis. State clearly which scenario your results supported. From the data, it’s clear that specificity prevailed (very high divergence, low overlap). Perhaps mention what is known about insect gut microbiomes in harsh environments or in detritivores. It’s known that many detritivorous insects rely on a limited set of gut symbionts (or sometimes none obligate). If chironomids harbour fewer ASVs, this fits the idea that many insects have relatively simple microbiomes (line 244-248 you stated that). You can cite general insect microbiome literature to say this finding of low diversity gut vs outside is consistent with insects filtering their microbiota or maintaining only certain bacteria. If any specific genera dominated chironomid guts in your data, discuss them.

ANSWER: We thank the reviewer for prompting a deeper elaboration of this aspect that we addressed. New parts of the text and references include the following

“The observation that chironomids have fewer sequence variants than their surrounding environment is not in contrast with ecological knowledge. There is compelling evidence supporting the idea that many detritivorous insects harbor a limited set of gut symbionts, and that the low bacterial diversity in their guts compared to the surrounding environment reflects selective filtering or maintenance of specific microbial communities. Insect guts often contain fewer microbial species than mammalian guts, and many insects rely on specialized bacterial communities for essential functions like digestion and defense. [Engel and Moran 2013]. A large-scale study analyzing gut microbiota across 218 insect species found that gut bacterial diversity is significantly lower than environmental microbial diversity, and that omnivorous and detritivorous insects tend to host fewer bacterial taxa. Gut microbiota composition is shaped by host diet, developmental stage, and phylogeny, reinforcing the role of host-driven selection [Yun et al., 2014]. “

The absence of differences between chironomid species or sites is a noteworthy finding, as one might expect different species (especially those with different feeding habits) or different glacial sites (with different conditions) to harbour different microbiomes. The fact that this is not the case suggests a strong commonality. Discuss possible reasons:
- Could it be that all these chironomid species feed on very similar resources (e.g., microbial mats, detritus) in the glacier streams, thus acquiring a similar microbiome?

- Or perhaps the host filters the microbiome so strongly that regardless of environment, only certain bacteria can thrive in the gut (due to physiological conditions like anoxia, antimicrobial enzymes, etc.).

- It might also be partially methodological: if each species had few samples, maybe differences weren’t detectable – if so, mention that sample size could be a limiting factor (but don’t undermine your result too much; just a cautious note if appropriate).

- If any species were aquatic vs semi-terrestrial, could that matter? (Likely all larvae were aquatic, though.)

As concerns feeding habits,all the species that we collected are detritivorous/scrapers. None belongs to predators. These taxa are also all bound to the high-mountain cold-water environments. Only *Metriocnemus* genus includes semi-terrestrial species. The genus occurs in one of the widest biotope ranges of any dipteran genus (Sæther 1989), including from mosses and higher vegetation, pitcher plants and hollow trees to margins of springs, ditches, streams, damp soils, and hygropetric biotopes, and occasionally lakes and rock pools (Cranston and Judd 1987; Sæther 1989, 1995).

Cite implications and future directions.

ANSWER: The following concepts, in reply to a similar comment from Reviewer 2, have been added to the text in the conclusive section: “The implications of the present results for glacier ecology and chironomid biology are that these symbiotic bacteria may protect these insects from environmental stressors, even of natural origin (e.g. extremely low temperatures), by promoting the digestion of food with low nutritional value and/or contributing to the insect’s health and fitness through participation in its metabolism. Knowing more about the array of hosted microbiota could support studies devoted to the transmission of potentially critical microbial taxa from adult to egg to hatched offspring, providing a more advanced understanding of their mutual relevance in fragile and endangered ecosystems.”

Figure 1: Scale bar and north indication are missing. I would remove the black background.

ANSWER: The figure has been edited to address these and other issues, including the substitution of the copyrighted satellite imagery with a non-photographic map. Scale bar and North direction arrow have been added, and the black background has been removed.

Figure 2: Explain all colours in the caption. Word colours and taxa and abund gradient.

ANSWER: The requested specifications have been added to the legend

Figure 3: Chao1 axes name bracket is missing. Statistic is missing.

ANSWER: The bracket has been closed. And the statistics added to the text (“The overall alpha diversity statistical difference, following a Welch ANOVA test gave an F-value of 42.75 and a strongly significant p value of 3.945e^-11^. The individual pairwise Wilcoxon Rank Sum post hoc comparison provided the following differences: insect vs. pond: 0.011061; insect vs. glacier: 0.014671; pond vs. glacier: 0.8598 ( p-values based on the Benjamini-Hochberg False Discovery Rate correction.“).

Figure 4: Not necessary, the table in the text looks better.

ANSWER: The figure has been moved out of the manuscript body (see query above).

**Reviewer #2:**

Dear Authors
Thank you for the effort you have put in this paper. This manuscript provides original insights into the ecological separation between glacier microbial communities and insect microbiomes, which are highly relevant in the context of global warming and glacial retreat.
I have some concern, as it needs some improvements:

ANSWER: We thank the reviewer for remarking on the effort and commenting on the originality and relevance of the resulting data.

*Enrich the introduction or discussion with Important biological and morphological aspects specific to the species in question. This may facilitate the interpretation of the findings.

ANSWER: The introduction and the discussion have been revised, adding details on the insects.

*Use Well-drawn speculative interpretations to avoid firm conclusions.

*better statistical support for key results, especially for beta diversity (e.g., PERMANOVA9.
*Better visualization for beta diversity (NMDS maybe).

ANSWER: For this very important aspect that has been signaled by both reviewers and extensively acted upon, we paste the answer provided above.

We have performed the statistical analysis recommended and added a new figure with the 3D ordination plot of the NMDS showing the separation between the insect- and environment-associated bacteria. The results were backed up by three statistical analyses (PERMANOVA, PERMDISP and ANOSIM) whose significant p values are shown in the same novel figure (New Fig. 4).

In addition, we have constructed the Venn diagram as suggested and included it in the Supplementary Material.

We have likewise computed the suggested index and added the values in the text as follows.

“The Jaccard similarity index on the whole ASV data matrix yielded the following values: insect vs. pond: 0.0564; insect vs. glacier: 0.0548; glacier vs. pond: 0.1336. As regards the Bray-Curtis distances those were: insect vs. pond: 0.9367; insect vs. glacier: 0.9412; glacier vs. pond: 0.5504.”

*Improve clarity by shortening some sentences or dividing them into smaller parts (See Below)

ANSWER: The wording and complexity have been deeply addressed throughout the manuscript

*the introduction is Simple and well-written, both cohesion and coherence.

ANSWER: We acknowledge this positive note. The introduction has now also been improved upon some specific comments from both reviewers.

*Line 26–28: avoid “to understand whether bacteria … could be considered food” (this phrasing implies a strong dietary hypothesis that the data don’t fully support).

ANSWER: We agree, but that is actually the result, while, when formulating the hypothesis, we did not have the data yet. In practice, the actual outcome was within the alternative possibilities. To clarify this open-ended premise, we added the words ‘or not’ to the sentence: *“…to understand whether or not bacteria …etc. “*

*Lines 45–50: Avoid conclusions like “data showed no clues,” as this will decrease the value of your work hypothesis. Consider using “suggests” or “provides limited support for…”

ANSWER: We agree, and we adopted the second suggested wording.

*Lines 53–59: shorten the sentences or divide them into shorter sentences to improve readability.

ANSWER: The sentences (now placed in a different part of the restructured introduction) have been revised and shortened.

*Lines 72–78: Avoid repetition and be more direct. This passage can be shortened to fewer lines

ANSWER: The text has been simplified and shortened

*Line 105–112: Try here to answer the question, why does this study matter? Why are you hypothesizing a confrontation between the Chironomidae gut and the Environmental Microbiome?

Why did you expect that the Chironomida diet could be directly from bacteria in its habitat?

ANSWER: There were premises to cast our hypotheses. Fair et al. (2024) suggested that they serve as keystone species in supraglacial meltwater environments. Microbial genera richness, indicator genera richness, and *Polaromonas* relative abundance declined with increasing larval Chironomidae abundance. Other reports indicated that bacterial composition within aquatic invertebrates varied with taxonomy, habitat, diet, and time of sample collection (Kroetsch et al., 2020). These considerations have been added to the introduction.

*Line 121–124: poor description of the hypothesis, and it would be better to be placed before the research question/objectives

ANSWER: The hypothesis description has been implemented by clarifying clauses and moved above the scopes as suggested. The description is now the following:

“The working hypothesis of this investigation was that there could be one of two possible statuses: A) Symmetry (high overlap between communities) i.e., the Chironomids’ microbiome mirrors that of the environment, as microbes are indiscriminately acquired; B) Specificity (high degree of divergence between communities), i.e., chironomids fractionate and select which bacteria would be established in their sequencing-detectable microbiome. We assumed that the prevailing scenario between these possibilities would be revealed when comparing the insect microbiota with its outer environmental counterpart.“

*More detailed information on the sampling protocol is needed to ensure reproducibility

ANSWER: The protocol was widely implemented with details. The text now reads as follows:

“Chironomid larvae were collected from their water habitats using a hand-held pond net fitted with a 100 µm mesh. Sampling was conducted during daylight hours. The net was gently swept through the water body. After each sweep, the contents of the net were immediately transferred into white plastic trays filled with site water to facilitate visual sorting. Chironomid larvae were manually isolated using fine forceps and placed into sterile 1.5 mL microcentrifuge tubes containing 95% ethanol for preservation. Taxonomic identification was initially done via a morphological approach (microscopic examination of larval features). When morphology was not enough for an appropriate identification, we performed molecular identification of the insect by extracting the larval DNA and amplifying the mitochondrial cytochrome oxidase I (COI) gene, following the protocol of [34]. Briefly, genomic DNA was extracted from the larval body using a QIAGEN DNAeasy Blood & Tissue Kit, following a slightly modified protocol for the purification of total DNA from animal tissue. In particular, 10 μl of a 1 M DTT (dithiothreitol) solution were added to enhance the removal of chitin, after which the samples were incubated overnight at 56 °C for the initial lysis step. The final elution was performed in 50 μl of AE buffer to increase the final DNA concentration. The mitochondrial (COI gene were amplified and sequenced using the primers LCO1490 GGTCAACAAATCATAAAGATATTGG and HCO2198 TAACTTCAGGGTGACCAAAAAATCA.

Adult chironomids were occasionally encountered, and two specimens, collected by tweezers, one on the Mandrone glacier and one on the Amola glacier (Tab. 2) were included in the analysis. Environmental samples were obtained by collecting material directly into 50 ml sterile Falcon tubes, except the slime on submerged stones, from which the material was removed by scraping with a fine brush and suspended in 20 ml of physiological sterile solution.

In total, the collected material included 36 larvae, 2 adults. and 12 environmental samples.”

”

*Good coverage of multiple glacier systems.

ANSWER: We appreciate this positive remark

*Line 129: report how many larvae here also.

ANSWER: The numbers have been reported at the end of the sampling protocol description (see text two queries above)

*Line 151: Why were adults included, and also why did you base our work on larvae?

ANSWER: The adults are here as a marginal extra. It is very rare to encounter any since they are very short-lived, they die soon after mating, they do not fly unless in the absence of wind, they do not live in contact with the same (aquatic) environment where larvae are found, and they tend not to feed, or to do it on very different sources as plant pollen. Encountering two of them has therefore been mere luck. We included those in the analysis as they became available and offered a chance to view (although without statistical support) whether their microbiome would display some clues of particular differences. Some authors have reported an expected decline in microbiome diversity throughout the different developmental stages from egg to larva and to adult in *Chironomus transvalensis* (Sela et al., 2019). Larvae are instead the stage that represents most of the chironomids’ life cycle timewise and the form that has a substantial interaction with the environment.

We have added some of these specifications to the text.

*Line 192: and beta diversity ??

*Line 230–232: Clusterings are described, but some statistical evidence and significance (e.g., p-values or clustering robustness, PERMANOVA).

ANSWER: Both these aspects have been addressed with the new statistical data and the novel figure with the beta diversity NMDS plot (see above answer to the third and fourth query.

*Line 239-242: Again, what was the purpose of using adults?

ANSWER: see the answer to the two queries above (comment about line 151)

*I would like to see here a PCA or NMDS with a PERMANOVA plot for beta diversity. It would be more representative and clearer than a dendrogram

ANSWER: As above, NMDS, PERMANOVA, ANOSIM, and PERMDISP results are provided in the new figure.

Lines 316–321, 394–396, do you think that rapid digestion or DNA fast degradation may have a role in the observed result? (For the discussion)

ANSWER: Indeed, this aspect could be relevant, and we thank the reviewer for raising it. We have added those considerations to the text.

Lines 467–471: The discussion of atmospheric deposition as a driver of high bacterial richness in glacial water is speculative and not supported by any direct evidence in this study. It should be introduced more as an explanation.

ANSWER: As a matter of fact, we had intended that issue not as specifically tied to the sites of this investigation but rather as a global background aspect. To clarify this, we have added literature reporting the quantitative data. Bacterial deposition rates can range from 0.3 × 10⁷ to 8 × 10⁷ cells/m²/day (Reche et al., 2018). The estimated global emission rates of bacteria that fall back on our planet amount between 0.7–28.1 Tg/year. (Woo and Yamamoto, 2020)

Lines 490–495: Avoid making definitive conclusions. Rephrase to acknowledge limitations of 16S metabarcoding in identifying food-derived taxa.

ANSWER: We have toned down the assertions by changing “*yielded a convincingly clear answer*” with: “*evidence has been found*” and “ *states against, and allows one to reject that assumption*“ with: “*renders that assumption unlikely.*”

In conclusion, is the similarity of 4-5 % a good indicator of similarity or not? How much is good similarity? Does similarity mean direct feeding behavior and diet preference, or is it a coincidence or even indirect?

ANSWER: A less than 5% similarity is objectively a low proportion. If the microbial flow from the environment into the consumer were a straight projection of the external assemblage, the dominant members of both communities would coincide, ruling out coincidental effects. We have added this consideration to the text.

For that, in the introduction, it would be helpful if you add some morphological aspects of Chironomidae, such as feeding behaviour and mouth parts for both larvae and adults. From where you start your hypothesis? are we assuming that the Chironomid feeds on soil? OM? Slure?..rthis aspetc must be discussed

Is this a sign of feeding behavior, perhaps they like, or choose voluntarily which microbiome to feed on?

ANSWER: We have implemented the description as follows:

“ The dietary habits of chironomids are influenced by the type and quality of food present in their environment. For instance, habitats rich in epiphyton or sandy areas tend to support more algae, while organic sediments offer higher levels of detritus. Most chironomid larvae reside at the bottom, living in niches from which they extract nutrients suspended in the water. Some species forage by scraping or gathering organic matter from sediments or rocky surfaces in lakes and rivers. A few are carnivorous, preying on other chironomid larvae (Lencioni et al., 2024). As adults, chironomids consume substances that contain sugars such as sucrose and glucose (Burtt et al., 1986).”

Fig3. I would use the same order of groups in B as in A (insect, pond, and finally, glacier).

ANSWER: The parts have been reordered as suggested.

Line 361- and 491-: table 4 Did you check if any of these unique Chironomid gut microbiome taxa are associated with environmental studies in the area or nearby in other studies?
Or
*Did you check at least their ecological groups? Can they be free in the environment, or must they have a host? in which environments they drive better?
*For example, it is surprising that Janthinobacterium, which are commonly found in glaciers and lakes, are not present in the environmental samples here, but instead in the gut.

ANSWER: First of all, we are grateful to the reviewer for spotting the *Janthinobacterium* case, which led to an inspection of the whole table and to find out an error borne by an Excel formula (the filtering for ‘presence zero’ of ASV in either the insect or in the habitat sample had not carried the clause of including the minority of ASVs that were in both before extracting unique and duplicate names). Its correction led to some slight changes in the list, and, indeed, there were some *Janthinobacterium* entries featured by the habitat and insects as well. Their abundance in the insects was, however, always much higher, with a mean 15-fold enrichment compared to the habitat. In correcting the table, we also chose to adopt a taxonomical refinement with respect to the crude pipeline bioinformatics output. Those tools routinely yield lineages in which missing values are ‘filled’ by their higher rank name. Therefore, there could be phyla names that are carried all over and repeated even through the genus denomination instead of leaving the blank/undetermined field. For this reason, in listing the orders, families, and genera in the unique cases table, we reported only those that correctly featured the rank suffix (-ales, for orders, -aceae for families) and the proper nominative case nouns for genera.

As concerns the first part of the query, we have implemented the text with the following and added 19 new references (Note, since the Results and the Discussion sections have been joined in a single section, these parts are now located in the sixth paragraph of the combined section.

“Regarding the lineages that were uniquely encountered and are shown in Tab. 5, the following comments can be made on their identities. The archaeal phylum Crenarchaeota has been found in cold environments, including retreating glacier soils [63]. They are known for ammonia oxidation and are often detected in environmental DNA surveys of cold habitats. Margulisbacteria are siblings of Cyanobacteria with a hydrogen-based metabolism and can be found in harsh environments [64]. Sumerleota and. Hydrogenedentes are associated with the subsurface biosphere, are involved in sulfur and nitrogen cycling, and are adapted to anoxic conditions [65]. A similar case can be made for Caldisericota, which are more thermophilic and anaerobic, and are found in geothermal and subsurface environments, but have also been signalled in glacier environments [66]. At the genus level, *Providencia* has also been retrieved from insect guts as an opportunistic pathogen and in aquatic environments [67]. *Veillonella* is an anaerobic taxon that is found in gut microbiomes including insects [68]. *Leptotrichia* is a host-associated bacterium found also in the microbiomes of insects [69]. *Yokenella* is encountered also in aquatic insects and is gut-associated [70]. *Xenorhabdus* is reported to be symbiotic with nematodes, but pathogenic in insect guts [71]. *Phascolarctobacterium*, instead, is reported to be a succinate fermenter in insect-resident microbiota [72]. Many of the habitat-specific genera are ecologically coherent, playing roles in sulphur cycling, and syntrophy. They are often free-living in glacial environments and include *Sulfuricella* [73], *Smithella* [74]. Extremophiles as *Thermobacillus* [75] or *Geobacillus* [76] and *Hydrogenophilus* [77] are instead reported as cases of thermophiles that are nevertheless found within cold contexts as permafrost, occurring also in Antarctica and Himalaya. Other taxa that are only found in environmental samples are known to be cyanobacteria, which can contribute to primary production in water and epilithic habitats. These include *Geminocystis*[78] , *Symphothece* [79], *Nodosilinea* [80] and *Aliterella* [81].“

* Did you check for any seasonal impact? *When did you perform the sampling?

ANSWER: All sampling dates are shown in the second column of Table 1. The campaigns took place in the summer months (July-August-September) as this is imposed by the chironomids’ life cycle in these high mountain glacial habitats, which restricts their presence to a limited window of the year. Consequently, seasonal shifts cannot be investigated.

*Other genera are common in thermophilic environments, thus did you check the life cycle of the Chironomid?

*Are they migratory, or long-distance travelers?

*How long do adults live? How long does it take from adult hatching to laying eggs?

*They are present in glaciers, but is it their preferred habitat?

ANSWER: The chironomids investigated in this research are all cold stenothermal species. Within these only *Diamesa steinboecki* is exclusive of glacier-fed streams, but also the others are nevertheless all bound to high-mountain water habitats. The length of the life cycle is species-specific and can vary according to the local climatic conditions (e.g., the duration of the winter period and, therefore, that of the “biological window”). These species are neither migratory nor long-distance travelers. They fly rather poorly and only in the absence of wind. The dispersion capacity is low (a few hundred meters even if on the glaciers and in their proximity). They have reduced wing size and mate on the ground due to strong winds.

*Line 459: For more academic tone, change “In the last few years, it has become clear…” to “Recent studies have increasingly shown that…”

ANSWER: (?) Those words were actually not present in our manuscript (neither at line 459 nor elsewhere).

*Line 515–519: The moonmilk beetle comparison is interesting but need more clarification, is it relevance to glacier ecosystems or insect ecology.

ANSWER: We added a clarifying link to that topic: “The common aspect is inferring the food source of insects living in stressful, low-productivity habitats.”

*Line 498: “we definitely conclude” is too strong, replace with “we propose” or “we interpret”.

ANSWER: (?) Also in this case, those words were actually not present in our manuscript (neither at line 498 nor elsewhere).

*Lines 527–530: The sentence structure is long and dense. Consider breaking it.

ANSWER: The sentence has been broken into two shorter ones separated by a semicolon.

The closure of the discussion misses:

*What does this mean for glacier ecology or chironomid biology

ANSWER: The implications of the present results for glacier ecology and chironomid biology could involve the possibility that these symbiotic bacteria may protect these insects from environmental stressors, even of natural origin (e.g. extremely cold temperature), promoting the digestion of food with low nutritional value and/or contributing to the insect’s health and fitness by co-participating to its metabolism. These concepts have been added to the text.

*How could these findings be extended (e.g., functional profiling, manipulation of microbiomes, longitudinal studies)

ANSWER: Knowing more details about the array of hosted microbiota could support studies devoted to the transmission of potentially critical microbial taxa from adult to egg to hatched offspring that entail a more advanced comprehension of their mutual relevance in fragile and endangered ecosystems. This part has also been added to the conclusive remarks.

Hoping to have fulfilled all the pending issue, we thank you for your editorial work and kind attention.
