# Supplementary material for: High microbial diversity in glacial habitats uncoupled from the specialized microbiomes of resident chironomid fauna
Source: PLoS One. 2026 Jan 8;21(1):e0340651. doi: 10.1371/journal.pone.0340651 (PMC12782408; doi:10.1371/journal.pone.0340651)
Supplement: Attachment — Submitted filename: Answers to Reviewers second round.docx [file pone.0340651.s004.docx]

**Reviewer #1**:

I thank the authors for having considered all the suggested revisions and for their substantial work on the manuscript. I just have a couple of additional recommendations.

Lines 200–206: This subsection is very short. I suggest merging it with the end of the previous one.

ANSWER: done

Figure 1: The resolution is very low. Even when downloading the high-resolution file, the two maps appear blurry. I suggest recreating it using, for example, QGIS, which allows the creation of high-resolution satellite maps.

ANSWER: The image resolution aspect has been fixed by re-acquiring it from the source chart. See new the new version of Fig.1.

Figure 4: I recommend keeping a white background, as in the other figures, to maintain consistency throughout the article.

ANSWER: Fig. 4 has been remade using the with background as suggested.

Figure 5: The species names should be in italics.

ANSWER: The names have been rewritten in italics.

Figures 5 and 6: The color description of the cells is missing in the caption, as done in Figure 2 (“The cells reporting the numbers of taxa and the read abundance are colored by the Microsoft Excel conditional formatting gradient from red (lowest numerical values) to green (highest numerical values)").

ANSWER: The legends have been edited to include the recommended explanations.

**Reviewer #2:**

Dear Authors:
Thank you for the revised manuscript. Most of the previous comments have been addressed, and the responses were helpful. A few minor points remain:

• The text flow has improved, but some long and complex sentences remain, particularly in the discussion.
• Grammar and syntax have improved but are still somewhat dense in places.
• Line 26:28 The sentence has only been partly revised; it still implies diet. This should be fully rephrased.

ANSWER: As explained in the previous round, we totally agree, but the referral to a diet function is not stated as a result but as one of the initial hypotheses (that the data ruled out as we repeatedly state throughout the paper). Honestly, we had taken into account also that hypothesis, since in such a primary production-limited habitat, speculating that aquatic larvae could graze over bacterial biofilm slime and live upon that source of carbon and energy was, in our opinion, a legitimate possibility to test.

Therefore, when , in the prior round of revision, we rewrote the mentioned sentence in the following form ”*We aimed to assess the extent to which insect-borne bacteria resemble those found in icemelt water and the surrounding wet and terrestrial environments, in order to determine also whether or not bacteria in the external habitat could serve as food for these animals.*” we simply expressed the truth of our initial reasoning.

Anyhow, there is no problem in modifying the sentence and avoiding any mention to a diet function and for this purpose we have changed the sentence as follows:

“We aimed to assess the extent to which insect-borne bacteria resemble those found in icemelt water and the surrounding wet and terrestrial environments, in order to determine also whether the bacteria found associated with the insects could be interpreted mainly as specific dwellers, putatively involved with active physiological functions, or also as transient cells taken in for other purposes. “

• While biological interpretation has been improved, the ecological implications could be explored further for example, how microbiome specificity might affect resilience to glacial melt or colonization of new environments
• Ecological roles of taxa remain speculative.
Overall, the manuscript has improved, but addressing these remaining issues would further strengthen clarity and scientific rigor.

ANSWER: We thank the reviewer for requesting further elaboration on the ecological implications of our findings. We have leveraged the high degree of microbial specificity we observed to provide a more definitive interpretation of the functional roles of the dominant taxa within the chironomid core microbiome and the surrounding environment.

We have added the following text and constructed three new tables, placed in the supplemementary information file:

“The finding of a highly specific, low-diversity chironomid microbiome, which is strongly uncoupled from the diverse external community (only 4.9% shared ASVs, with insect communities having sevenfold lower diversity), suggests a critical host-driven selection process. A stable, specialized core community has significant ecological implications for the host's fitness in the face of glacial retreat and environmental change. These involve: (a) resilience to glacial melt; (b) colonization of new environments. A table, outlining the mechanisms by which microbes can be of support in these actions and the ensuing consideration is provided in the Supporting Information Table S1”

| **Ecological concept** | **Suggested mechanism of microbial support** | **Specific ecological insight** |
| --- | --- | --- |
| Resilience to glacial melt | Metabolic Stability and Detoxification: Glacial melt introduces environmental instability, including fluctuating flow rates, temperature spikes, and chemical shifts. The highly specific core microbiome acts as a physiological buffer. Taxa like *Providencia* and *Serratia* (core insect taxa) are functionally known in insect systems for their roles in detoxification and breaking down complex or inhibitory compounds, ensuring metabolic continuity even when the ingested detrital quality rapidly changes due to melt events. | This stable internal community can confer resistance to environmental stress, allowing the chironomids to maintain essential physiological functions where a generalist community would fail to cope with fluctuations typical of glacier-fed streams. |
| Colonization of new environments | Nutrient Supplementation (The 'Symbiotic Propagule'): The lack of significant difference in microbial community structure across ten chironomid species and three distant glacier sites underscores the perspective importance of the core microbiome. This core community could entail also a vital symbiotic propagule that the host has an advantage in carrying with it. | By supplying essential nutrients in a consistent manner, such as nitrogen or complex carbon resources, this core could facilitate the rapid and successful colonization of newly deglaciated, oligotrophic, primary succession habitats. The host-symbiont pairing seems pre-adapted to the nutrient-scarce conditions inherent in these environments. |

**Tab. S1.** **ecological implications expected for the microbiota of the investigated envirooinment**

“As regards the possible ecological roles of the observed taxa, these remain speculative because functional roles derived from 16S rRNA gene data are inferences, and not confirmed activities. However, the consistent and unique enrichment of specific bacterial taxa within the insect gut (constituting almost 25% of strictly insect-specific taxa) supports their functional necessity. Their known metabolic capabilities, when linked to the host's ecological niche (detritivory in oligotrophic, cold environments), provide circumstantial evoidences for their roles. The dominant, recurring members of the insect core group are *Providencia*, *Serratia*, *Massili*a, and *Flavobacterium*. Their likely exerted ecological roles have been listed and commented in Supporting Information tab. S2.”

| **Dominant core taxon** | **Inferred ecological role in chironomid gut** | **Insights** |
| --- | --- | --- |
| *Providencia, Serratia* (Gammaproteobacteria) | Nutrient Scavenging and Detritivory: These genera are commonly associated with insect guts and are known for their high metabolic versatility, including the breakdown of complex organic compounds. In the low-quality, detritus-rich diet of the chironomid, they are likely key primary decomposers, providing the host with readily absorbable organic acids and amino acids. | Their prevalence suggests a dedicated function in maximizing energy and nutrient extraction from a nutritionally poor food source, which is crucial for survival in the oligotrophic glacial stream environment. |
| *Massilia* (Betaproteobacteria) | Cold-Adaptation and Immune Support: *Massilia* members are frequently psychrotolerant (cold-enduring) and have been implicated in various insect-microbe interactions. They likely contribute to host fitness by maintaining robust metabolic activity at the low temperatures typical of glacier-fed streams. | Their role is inferred to be one of physiological support, maintaining gut homeostasis and potentially participating in defense or signaling under extremely cold conditions. |
| *Flavobacterium* (Bacteroidetes) | Polymer Degradation (e.g., Chitin): Members of the Bacteroidetes phylum are renowned for their massive arsenal of glycoside hydrolases. *Flavobacterium* is likely selected for its capacity to break down large, tough biopolymers, such as the chitin found in fungal debris or the cell walls of microbial mats that form part of the chironomid's scraped or ingested diet. | This is a highly specific enzymatic service required to release sequestered nutrients and energy that the chironomid host could not obtain otherwise, further suggesting a direct role in nutrition. |

**Tab. S2.** **Ecologically insightful roles of insect-specific core taxa**

“Conversely, for the corresponding inference regarding the taxa that dominate the surrounding habitat, parallel considerations are outlined in Supporting Information Tab. S3.”

| **Dominant environmental taxon/group** | **Inferred ecological role in habitat** | **Insights** |
| --- | --- | --- |
| *Arenimonas* | Primary Colonization and Nutrient Mineralization: Often found in proglacial sediments and newly deglaciated soils. | This genus is a key pioneer colonizer, capable of metabolizing diverse organic compounds and mineralizing phosphorus and nitrogen, which facilitates the early stages of ecosystem development. |
| *Methylotenera* | Carbon Cycling (Methylotrophy): These are specialized bacteria that utilize one-carbon compounds (e.g., methanol, methylamine). | They act as critical carbon processors in the habitat, often forming syntrophic relationships that mitigate the release of methane or process specialized carbon sources available in glacial meltwater. |
| Nitrosomonadaceae | Nitrogen Cycling: , members of this family are recognized chemoautotrophs, specifically involved in nitrite oxidation (a key step in nitrification). | Their role solidifies the glacier habitat as a site of intense, externally-driven nitrogen transformation, but the lack of strong overlap with the insect gut suggests that the chironomid's core microbiome is selected to bypass reliance on this external cycle. |
| Vicinamibacterales | Sorption and Adherence: This order often includes taxa associated with soil and sediment, likely serving a role in binding and forming the microbial mats and biofilms from which the chironomid larvae forage. | Their high prevalence highlights the structural microbial component of the glacial environment, which forms the physical substrate that the chironomids scrape or ingest, but their limited presence in the gut suggests effective host selection/filtering. |

**Tab. S3.** **Ecological insights into environment-dominant taxa**

Hoping to have fulfilled all the pending issues, we thank you for your editorial work and kind attention.
